# Biodegradation of Polyhydroxybutyrate, Polylactide, and Their Blends by Microorganisms, Including Antarctic Species: Insights from Weight Loss, XRD, and Thermal Studies

**DOI:** 10.3390/polym17050675

**Published:** 2025-03-02

**Authors:** Volodymyr Skorokhoda, Ihor Semeniuk, Taras Peretyatko, Viktoria Kochubei, Oleksandr Ivanukh, Yuriy Melnyk, Yurij Stetsyshyn

**Affiliations:** 1 Department of Chemical Technology of Plastics Processing, Lviv Polytechnic National University, 3/4 St. George’s Sq., 79013 Lviv, Ukraine; vskorohoda@yahoo.com (V.S.); oleksandr.o.ivanukh@lpnu.ua (O.I.); yuriy.ya.melnyk@lpnu.ua (Y.M.); 2 Department of Physical Chemistry of Fossil Fuels of the InPOCC Named After L. M. Lytvynenko of the NAS of Ukraine, 3A Naukova Str., 79060 Lviv, Ukraine; igorsem777@gmail.com; 3 Department of Microbiology, Ivan Franko National University of Lviv, 4 Hrushevskogo Str., 79005 Lviv, Ukraine; taras.peretyatko@gmail.com; 4 National Antarctic Scientific Centre, 16, Tarasa Shevchenko Blvd, 02000 Kyiv, Ukraine; 5 Department of Physical, Analytical and General Chemistry, Lviv Polytechnic National University, 3/4 St. George’s Sq., 79013 Lviv, Ukraine; viktoriia.v.kochubei@lpnu.ua; 6 Department of Organic Chemistry, Lviv Polytechnic National University, 3/4 St. George’s Sq., 79013 Lviv, Ukraine

**Keywords:** biodegradable polymers, polyhydroxybutyrate, polylactide, microbial degradation

## Abstract

This study explores the biodegradation of polyhydroxybutyrate (PHB), polylactide (PLA), and their blends by 11 bacterial species (including Antarctic strains) and 6 fungal species. Aeration significantly enhanced PHB degradation by mold fungi (*Aspergillus oryzae*, *Penicillium chrysogenum*) and bacteria (*Paenibacillus tundrae*, *Bacillus mycoides*), while *Aspergillus awamori* was most effective under non-aerated conditions. For PLA, degradation peaked under aeration with *Penicillium chrysogenum* and *Bacillus subtilis*. PHB/PLA blends degraded slower overall, with maximum degradation under aeration by *Penicillium chrysogenum*, *Pseudoarthrobacter* sp., and *Flavobacterium* sp. Biodegradation was assessed via weight-loss measurements, X-ray diffraction (XRD), and thermal analysis. PHB samples showed reduced crystallinity and thermal stability linked to weight loss, while PLA samples exhibited varied changes, often with increased crystallinity and stability depending on the microorganism. PHB/PLA blends displayed variable crystallinity changes, generally decreasing under microbial action. The search for effective plastic-degrading microorganisms, particularly from extreme environments like Antarctica, is vital for addressing plastic pollution and advancing sustainable polymer degradation.

## 1. Introduction

Since the 1930s and 1940s, following the initial reports on the development of synthetic plastics, there has been a continuous annual increase in their production and consumption [1,2]. In 2050, the global production of primary plastics will reach 1100 million tons [3]. As of 2022, a staggering 59% of virgin plastic was discarded directly into the environment, with a mere 7.2% being recycled and reused [4].

The overwhelming majority of plastics originate from fossil hydrocarbons. The chemical inertness and stability of their molecules pose significant challenges, making their disposal in natural conditions either difficult or nearly impossible. As a result, this plastic has accumulated in all major terrestrial and aquatic ecosystems, and its resistance to decay is expected to persist for an extraordinarily long period [5].

To address the issue of environmental pollution caused by plastic, one effective and economically viable solution is the investigation of bioplastics as alternatives to non-degradable synthetic plastics [6]. Commonly encountered types of bioplastics include poly(butylene succinate) (PBS), polyhydroxyalkanoate (PHA), poly(ε-caprolactone) (PCL), poly(butylene adipate co-terephthalate (PBAT), and others. Notably, polylactic acid (PLA) and other biopolymers derived from bacterial fermentations, such as poly(hydroxybutyrate) (PHB), have garnered significant attention and exhibit substantial potential for application in various fields due to their favorable thermal and mechanical properties [7,8,9].

Nevertheless, the utility of PLA and PHB is constrained by factors such as brittleness, poor plasticity, and relatively low thermal stability stemming from the minimal distinction between their melting and decomposition temperatures. A promising avenue for overcoming these limitations involves blending these two chemically similar biopolyethers, offering the potential for the development of new environmentally compatible materials with enhanced transport and mechanical characteristics [10,11,12,13]. Such composite materials are not only more degradable but also more cost-effective when compared to PHB-based materials [14]. Incorporating PHB into PLA films has demonstrated a noteworthy enhancement in oxygen barrier properties while concomitantly reducing wettability, rendering them effective for applications in food packaging [15]. The diverse methods employed for obtaining these compositions play a pivotal role in determining their structure, properties, and consequently, their applicable fields.

Due to their biodegradability and biocompatibility, PHB and PLA are extensively employed across various industries. In biomedicine, PHB is utilized for manufacturing suture threads, tissue culture supports for implants, surgical implants, dressing materials, bone components, vein replants, heart valve designs, and pins. It also plays a crucial role in pharmacology and veterinary medicine, where it is employed for encapsulating medicines to enable controlled release. In the food industry, PHB is used for product packaging, while in agriculture it finds application in the encapsulation of fertilizers. Furthermore, in environmental protection, PHB is instrumental in the production of bags, bottles, disposable items, personal hygiene products, biodegradable diapers, and the reclamation of areas affected by oil spills [16]. PLA stands out as a promising biomaterial with wide-ranging applications in various healthcare domains. Notably, it is utilized in tissue engineering for crafting cardiovascular implants, in dentistry for drug delivery, orthopedic interventions, cancer therapy, and skin and tendon healing. PLA’s significance extends to the manufacture of medical instruments and equipment. Its pivotal role as a 3D-printable biopolymer has been underscored, particularly during the global COVID-19 pandemic, emphasizing its versatility and adaptability in responding to pressing healthcare needs [17].

Irrespective of the designated application of biopolymers, comprehending, enhancing, and regulating their degradation is of paramount importance. Among the most prevalent polyhydroxyalkanoates is PHB, typically derived industrially from sugars or through microbial synthesis. The degradation of PHB is facilitated by microorganisms, involving both intracellular and extracellular hydrolases, categorized as PHA depolymerases (PhaZs, EC 3.1.1.75) [18]. Extracellular PhaZs play a crucial role in hydrolyzing semicrystalline PHA or amorphous PHB [19], breaking them down into oligomers and monomers that serve as a carbon source for microorganisms. In aerobic conditions, these oligomers and monomers undergo microbial metabolism, resulting in the production of carbon dioxide and water. Conversely, under anaerobic conditions, the transformation yields methane and water [20,21]. The rate of PHB degradation, and consequently its duration, is contingent on factors such as enzyme sequence, conformation, and concentration, in addition to environmental conditions and the inherent properties of the polymer itself. PHB degradation may occur over a span of months in anaerobic wastewater or extend to years in seawater, illustrating the variability influenced by these diverse factors [22].

The efficacy of PHB film degradation was assessed across nine bacterial strains, with five (*Comamonas testosteroni* 31A, *Cupriavidus* sp., *Paucimonas lemoignei*, *Pseudomonas stutzeri*, *Ralstonia* sp.) demonstrating confirmed PhaZ activity, and four (*Alteromonas macleodii*, *Loktanella vestfoldensis*, *Marinobacter alglicola* DG893, *Oceanibulbus indolifex* Hel45) exhibiting predicted activity. All bacterial strains with established PhaZ activity displayed the capability to degrade the film. Notably, among the bacteria with confirmed PhaZ activity, *Paucimonas lemoignei* induced a 12% mass loss in the film after 2 days, while *Cupriavidus* sp. led to a significant 90% loss after 4 days. Among bacteria with predicted PhaZ activity, Marinobacter algicola DG893 caused an 11% loss of PHB film mass after 2 weeks of growth. Among the strains with proven PhaZ activity, *Ralstonia* sp. showed the highest specific activity because less biomass was required for polymer degradation compared to other strains. *Ralstonia* sp. continued to decompose the film at pH 3.3–3.7 [22]. PLA stands out as a biodegradable and compostable thermoplastic material produced through the fermentation process of renewable resources [23]. Possessing properties similar to synthetic thermoplastics, PLA finds application as a packaging thermoplastic [24,25]. However, it is noteworthy that PLA is comparatively more challenging to decompose than other biodegradable plastics [26]. Microorganisms capable of degrading PLA are not as widely distributed in the environment when compared to those capable of degrading other types of plastics [27,28,29]. The high molecular weight of PLA retards microbial degradation in comparison to low-molecular-weight PLA [30]. Consequently, chemical hydrolysis takes precedence as the primary process for PLA depolymerization during the early stages of degradation, and it serves as the limiting factor for subsequent PLA biodegradation by microorganisms [31]. PLA exhibits decomposability under both aerobic and anaerobic conditions, particularly in elevated temperatures that facilitate rapid chemical hydrolysis [23,32,33,34]. PLA can undergo processing through mechanical and chemical methods [30]. Mechanical processing methods include grinding and processing waste PLA through melting; however, this can result in alterations to the mechanical properties of PLA [35,36]. On the other hand, the chemical processing method employs organic solvents to depolymerize PLA, yielding high-quality lactic acid. However, this approach involves expensive and intricate processes [35,37].

PLA in the environment undergoes chemical hydrolysis, breaking down into low molecular weight oligomers. These oligomers are subsequently completely mineralized by microorganisms’ enzymes to form carbon dioxide and water [38,39]. The degradation of PLA involves enzymes such as proteinase K, serine protease *Tritirachium album* [40], and alkaline protease [41]. Additionally, PLA depolymerase, synthesized by *Amycolatopsis* sp. K104-1, represents a type of protease specifically designed to hydrolyze PLA [39]. *Amycolatopsis* sp. bacteria, along with other strains such as *Bacillus smithii* [42], *Actinomudura* sp. [43], *Laceyella sacchari* [44], *Stenotrophomonas pavanii*, *Pseudomonas geniculata* [30], *Chryseobacterium* sp., *Sphingobacterium* sp., *Pseudomonas aeruginosa* [45], enzymatically hydrolyze PLA. The synthesis of protease and PLA-degrading enzymes is induced by the addition of gelatin, resulting in a significant increase in PLA biodegradation [30,38,46]. Actinobacteria that degrade PLA are primarily found within the *Pseudonocardiaceae* family, although representatives from the families *Micromonosporaceae, Streptomycetaceae*, *Streptosporangiaceae*, and *Thermomonosporaceae* have also demonstrated the ability to degrade PLA [47].

Inspired by previous research into the microbial degradation of polymeric materials, we conducted comprehensive biodegradation studies on PHB and PLA polymers, as well as their mixtures, using a diverse range of microorganisms (11 bacterial and 6 fungal species). Notably, this study is the first to explore the biodegradation of these polymers using pure microbial cultures. Special emphasis was placed on Antarctic bacterial strains due to their anticipated enhanced enzymatic activity toward the studied polymers under standard cultivation conditions. This aligns with findings from study [48], which identified 34 cold-adapted strains capable of degrading biodegradable plastics, underscoring the potential of novel microbial taxa for sustainable plastic recycling and their role in a circular plastic economy. Biodegradation in our study was assessed through weight-loss analysis post-cultivation, supplemented by XRD and thermal analyses to evaluate the impact of microbial activity on polymer structure and properties.

## 2. Materials and Methods

### 2.1. Materials

#### 2.1.1. Polymers

PHB was synthesized in the Department of Physical Chemistry of Fossil Fuels of the InPOCC named after L. M. Lytvynenko of the NASU. PLA Ingeo was obtained from NatureWorks (Plymouth, MN, USA).

Synthesis of polyhydroxyalkanoates. The bacterium *Azotobacter vinelandii* N-15 from the microorganism collection of the Department of Physical Chemistry of Fossil Fuels of the InPOCC named after L. M. Lytvynenko of the NASU, was used to produce PHB. An important factor in modern biotechnology is the selection of economically viable raw materials for culturing production strains. *A. vinelandii* N-15 was cultured in Ashby and Burke media in 750 mL Erlenmeyer flasks on a rotary shaker (220 rpm) at 30 ± 2 °C, with a pH of 6.9–7.2, for 48 h.

After 48 h, at the end of the stationary growth phase, the culture liquid was collected and centrifuged at 8000 rpm for 15 min. The supernatant was discarded, and the precipitate was washed twice with 0.9 wt.% NaCl solution, centrifuged for 15 min at 8000 rpm, and dried at 60 °C to a constant weight.

Extraction of PHB. PHB was extracted from the dry biomass of microorganisms by cell disintegration, chloroform extraction, and removal of cell debris, followed by precipitation with ethyl alcohol, isolation, and drying of the target product. Cell disintegration was performed by lysing the cells in ethanol and treating them with ultrasound (20 min at 30 °C) using a GT SONIC VGT-6250 ultrasonic bath (Meizhou, China). After centrifugation (15 min, 6000 rpm), the pellet was extracted in a Soxhlet apparatus with chloroform over five cycles. The resulting solution was concentrated, and the polymer was precipitated by adding methanol or ethanol, then isolated by centrifugation at 6000 rpm for 15 min. The polymer was purified by redissolving in chloroform and reprecipitating with ethanol. Solvent residues were removed by drying in a vacuum at 60 °C, and the purified PHB was stored in a desiccator over calcium chloride [49,50].

#### 2.1.2. Culture of the Microorganism

The six mold species and the eleven bacterial species (Table 1), including bacterial strains isolated from various substrates in the Antarctic marine environment [51,52,53,54,55], were stored in the Museum of the Department of Microbiology at Ivan Franko National University of Lviv and were used in this study.

### 2.2. Methods

#### 2.2.1. Polymer Film Production

Solutions of the individual polymers PHB, PLA, and the PHB/PLA (62/38) mixture were prepared by dissolving each polymer in chloroform (5% concentration) at 70 °C with stirring for one hour. The films were cast onto silicate glass plates placed on a horizontal surface using a universal applicator (AU1-65). The solvent was allowed to evaporate at room temperature over 48 h. Obtained films with a thickness of 100 ± 5 μm were further dried in a vacuum oven (SPT-200) at 60 °C for two hours [56].

#### 2.2.2. Media and Conditions for Cultivating Microorganisms

Mold fungi were cultivated in a modified Sabouraud medium containing 1% glucose, in 100 mL Erlenmeyer flasks sealed with cotton-gauze stoppers at 28 °C for 14 days. To 40 mL of Sabouraud medium, 200 μL of inoculum of a one-day microorganism culture and a polymer film were added. The polymer films were sterilized separately in Petri dishes by autoclaving at 1 atm before use.

Bacteria were cultivated in Trypticase Soy Broth (TSB) under standardized conditions: 100 mL Erlenmeyer flasks with cotton-gauze stoppers, at 28 °C for 14 days. To 40 mL of the medium, 200 µL of a one-day bacterial culture and a polymer film were added. Before use, the polymer films were sterilized separately in Petri dishes by autoclaving at 1 atm.

To investigate the effect of aeration on polymer degradation, the flasks were placed on a shaker operating at 300 rpm. After cultivation, samples were washed with distilled water, dried at 105 °C for six hours, and weighed. The degree of polymer degradation by microorganisms was assessed based on the weight loss of the sample after 14 days. Controls consisted of medium with polymer films but without microorganisms.

#### 2.2.3. Determination of Weight Loss

Degree of destruction (N), %

N = (∆m/m_initial_) · 100, (1)

where ∆m is the change in the weight of the polymer film after cultivation of microorganisms under certain conditions (or in the control under the same conditions of the study);

m_initial_ is the initial mass of the film before cultivation of microorganisms under certain conditions (or in the control under the same conditions of the study)

Degree of destruction compared to the control (n), times

n = N_exp_/N_c_, (2)

where N_exp_ is the degree of destruction of the polymer film after cultivation of microorganisms under certain conditions;

N_c_ is the degree of destruction of the polymer film in the control under the same conditions.

#### 2.2.4. X-Ray Diffraction Analysis (XRD)

**Figure 1:**
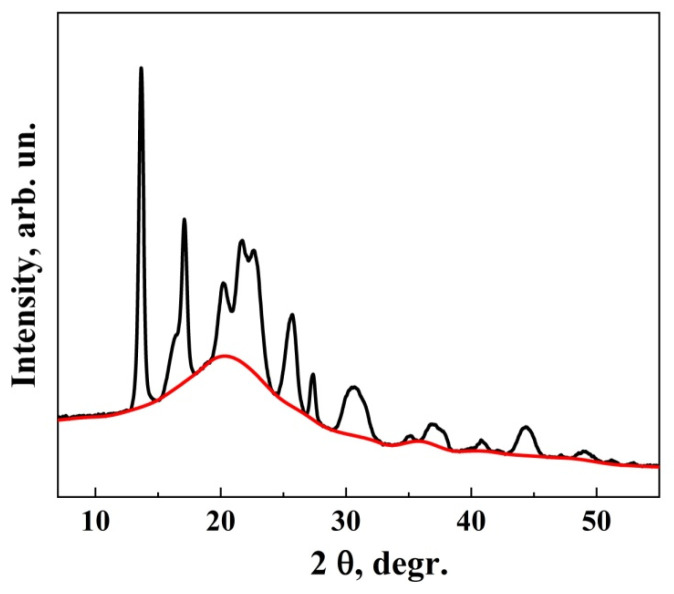
Sample of diffractogram with a “baseline” dividing it into crystalline peaks and an amorphous region. The red line shows the distribution between the amorphous and crystalline phases.

X-ray structural analysis was performed using an AERIS Research X-ray diffractometer (Malvern PANalytical, Malvern, UK). Polymer film samples were crushed and ground in an agate mortar. Diffraction patterns were recorded over the range of 7–55° (2θ) with a step size of 0.022° and a scan time of 24 s per step. To determine the degree of crystallinity of the samples, the data were processed using OriginPro software (OriginLab Corporation, Northampton, MA, USA). Spectra were smoothed and corrected for noise using the Savitzky–Golay filter. Crystallinity was calculated as the ratio of the integrated area under crystalline peaks to the total integrated area of the X-ray scattering curve. For this calculation, a baseline was drawn from the start to the end of the curve and through the bases of each peak (Figure 1).

This baseline divides the curve into crystalline and amorphous regions. The “Peak Analyzer” function was used to determine integrated area values for crystalline peaks and the overall curve. Crystallinity was calculated using the formula:

Crystallinity = Σ*A_c_*/Σ(*A_c_* + *A_a_*) (3)

where *A_c_* is the integral area under crystalline peaks, and *A_a_* is the integral area under the amorphous halo.

#### 2.2.5. Thermal Analyses (TGA and DTA)

Thermal analysis of samples was conducted on a Q-1500 derivatograph (Paulik-Paulik-Erdei system), connected to a personal computer. Samples were heated in an air atmosphere to 500 °C at a rate of 5 °C per minute, with sample masses between 35 and 40 mg. Aluminum oxide was used as a reference substance.

## 3. Results and Discussion

**Figure 2:**
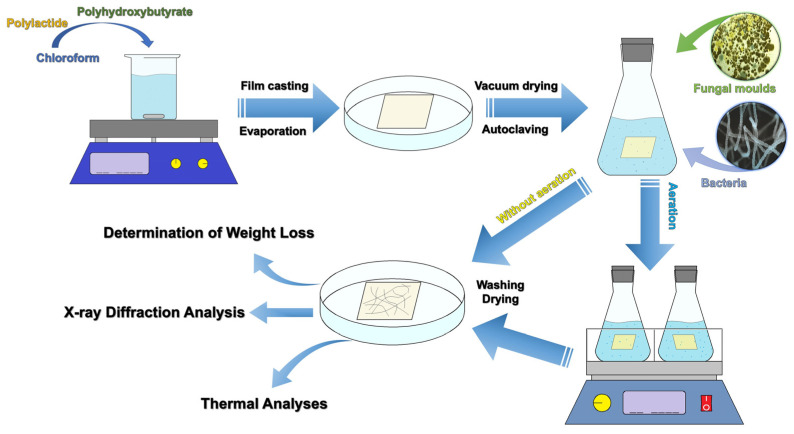
A general scheme illustrating the relationship between the preparation of polymer samples, their cultivation in a medium with microorganisms, and the analysis of their properties.

Our study is a complex, multi-stage process that includes the preparation of various polymer samples (polyhydroxybutyrate, polylactide, and blend films), their incubation with microorganisms, and the subsequent analysis of their properties after microbial exposure. The relationship between these stages is illustrated in Figure 2.

### 3.1. Mass Loss of the Polymer During Microorganism Cultivation

**Figure 3:**
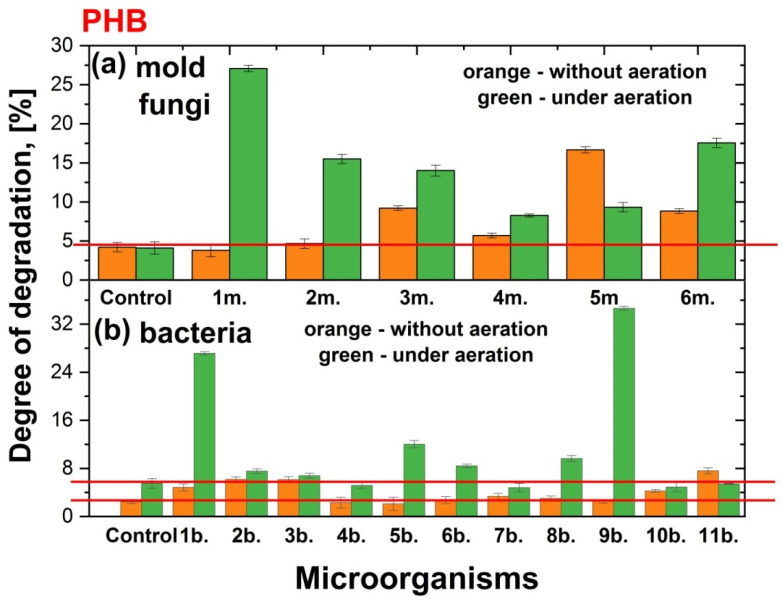
Degree of degradation (%) of PHB films without aeration (orange colour) and under aeration (green colour) after 14 days of cultivation in Sabouraud medium and by various mold fungi species (1m.—*Aspergillus oryzae*; 2m.—*Penicillium chrysogenum*; 3m.—*Trichoderma lignorum*; 4m.—*Aspergillus niger;* 5m.—*Aspergillus awamori*; 6m.—*Trichothecium roseum*) (**a**) and in TSB and by various bacteria species (1b.—*Paenibacillus tundrae* IMV B-7915; 2b.—*Pseudomonas yamanorum* IMV B-7916; 3b.—*Paenarthrobacter* sp. 28-in-78; 4b.—*Pseudoarthrobacter* sp. IMV B-7981; 5b.—*Flavobacterium* sp. 2B-in-99; 6b.—*Bacillus mesentericus*; 7b.—*Bacillus megaterium*; 8b.—*Bacillus cereus*; 9b.—*Bacillus mycoides*; 10b.—*Bacillus subtilis*; 11b.—*Streptomyces griseus*) (**b**). The red lines are a guide for the eyes.

**Figure 4:**
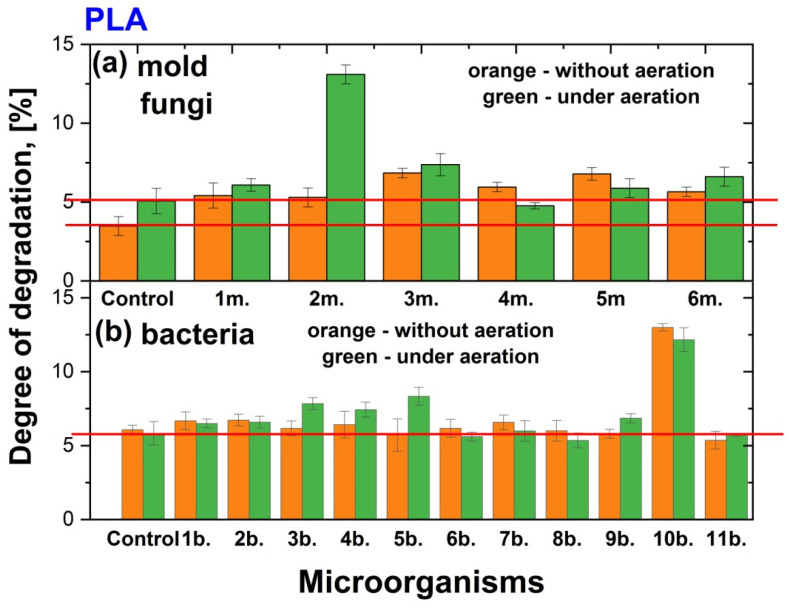
Degree of degradation (%) of PLA films without aeration (orange colour) and under aeration (green colour) after 14 days of cultivation in Sabouraud medium and by various mold fungi species (1m.—*Aspergillus oryzae*; 2m.—*Penicillium chrysogenum*; 3m.—*Trichoderma lignorum*; 4m.—*Aspergillus niger;* 5m.—*Aspergillus awamori*; 6m.—*Trichothecium roseum*) (**a**) and in TSB and by various bacteria species (1b.—*Paenibacillus tundrae* IMV B-7915; 2b.—*Pseudomonas yamanorum* IMV B-7916; 3b.—*Paenarthrobacter* sp. 28-in-*78*; 4b.—*Pseudoarthrobacter* sp. IMV B-7981; 5b.—*Flavobacterium* sp. 2B-in-99; 6b.—*Bacillus mesentericus*; 7b.—*Bacillus megaterium*; 8b.—*Bacillus cereus*; 9b.—*Bacillus mycoides*; 10b.—*Bacillus subtilis*; 11b.—*Streptomyces griseus*) (**b**). The red lines are a guide for the eyes.

**Figure 5:**
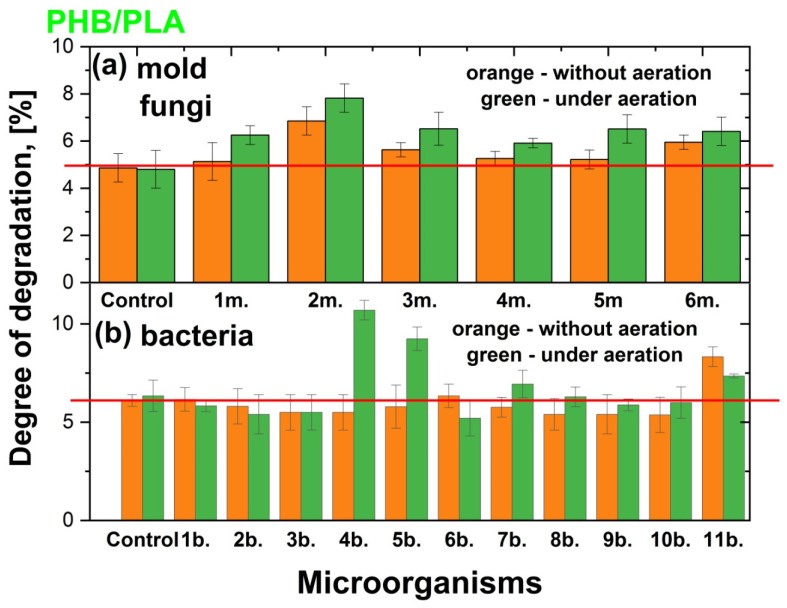
Degree of degradation (%) of PHB/PLA (62/38) films without aeration (orange colour) and under aeration (green colour) after 14 days of cultivation in Sabouraud medium and by various mold fungi species (1m.—*Aspergillus oryzae*; 2m.—*Penicillium chrysogenum*; 3m.—*Trichoderma lignorum*; 4m.—*Aspergillus niger;* 5m.—*Aspergillus awamori*; 6m.—*Trichothecium roseum*) (**a**) and in TSB and by various bacteria species (1b.—*Paenibacillus tundrae* IMV B-7915; 2b.—*Pseudomonas yamanorum* IMV B-7916; 3b.—*Paenarthrobacter* sp. 28-in-78; 4b.—*Pseudoarthrobacter* sp. IMV B-7981; 5b.—*Flavobacterium* sp. 2B-in-99; 6b.—*Bacillus mesentericus*; 7b.—*Bacillus megaterium*; 8b.—*Bacillus cereus*; 9b.—*Bacillus mycoides*; 10b.—*Bacillus subtilis*; 11b.—*Streptomyces griseus*) (**b**). The red lines are a guide for the eyes.

In this study, the degradation of films made from PHB, PLA, and a PHB/PLA blend by the mold fungi *Aspergillus oryzae*, *Penicillium chrysogenum*, *Trichoderma lignorum*, *Aspergillus niger*, *Aspergillus awamori*, and *Trichothecium roseum* was investigated under both static (without aeration) and aerated conditions. It is important to note that the degradation of polymer films also occurred without the influence of mold fungi, in the Sabouro medium, and in all cases, regardless of the chemical nature of the polymer films and the aeration of the medium or its absence, the degree of degradation was similar and amounted to about 4.5–5%. In turn, the degree of degradation of PHB, PLA, and PHB/PLA films by the bacteria *Paenibacillus tundrae* IMV B-7915, *Pseudomonas yamanorum* IMV B-7916, *Paenarthrobacter* sp. 28-in-78, *Pseudoarthrobacter* sp. IMV B-7981, *Flavobacterium* sp. 2B-in-99, *Bacillus mesentericus*, *Bacillus megaterium*, *Bacillus cereus*, *Bacillus mycoides*, *Bacillus subtilis*, and *Streptomyces griseus* was examined both under static conditions and with additional aeration at 300 rpm. In work [57] were shown degradation patterns of PHB, PLA or combined films at 37 °C in a phosphate buffer with a pH of approximately 7.4. The degradation rate was found to increase in the following order: PHB < PHB-PLA blend < PLA. In our case, after cultivation in trypticase soy broth we revealed a similar tendency. The degree of degradation of PHB was nearly 3% at stationary conditions and nearly 5% under additional aeration. At the same time, the degree of degradation for PLA and combined films as in stationary conditions such as under aeration was the same and around 5%. The impact of the microorgarganisms on the polymer film degradation was calculated and presented in two forms. First, the degree of destruction (N) in % was calculated by comparing the initial polymer mass of the sample with the mass of the same sample after cultivation in a nutrient medium or a nutrient medium with microorganisms (Figure 3, Figure 4 and Figure 5). Second, the degree of destruction (n) in times of polymer films by microorganisms compared to control obtained under the same conditions (Table 2).

#### 3.1.1. Impact of the Cultivation of Microorganisms on PHB Degradation

PHB has great potential in food packaging applications with better water vapor barrier properties than polypropylene and better oxygen barrier properties than both polyethene terephthalate and polypropylene [58,59]. Its great advantage is that it is obtained through biosynthesis, rapid degradation in the natural environment, as well as under the influence of microorganisms. As mentioned earlier, PHB was slightly degraded in Sabouraud medium and TSB without microorganisms during 14 days of thermostating, and while aeration had no effect on PHB degradation in Sabouraud medium, in TSB, aeration led to an increase in the degree of degradation almost twofold.

Among the studied species, the degree of PHB degradation under static conditions for mold fungi was significantly higher for *Trichoderma lignorum*, *Aspergillus awamori* and *Trichothecium roseum*. Notably, *Aspergillus awamori* was the most effective in degrading PHB films, achieving a degradation rate of 16.7%, which was 4.0 times higher than the control (Figure 3, Table 2). When aeration was introduced, the degradation rate for PHB by *Aspergillus awamori* increased only by 3.0 times compared to the control. At this time, the aeration had a notable impact on the degradation of PHB biofilms by mold fungi, particularly for *Aspergillus oryzae*, *Penicillium chrysogenum, Trichoderma lignorum*, *Aspergillus niger*, and *Trichothecium roseum*, with respective increases of 6.6, 3.8, 3.4, 2.0 and 4.3 times under aerated conditions compared to static conditions. Across all six mold fungi, additional aeration led to degradation rates that were 2.0 to 6.6 times higher than the control for PHB films.

Among the tested bacterial species, PHB degradation without aeration ranged from 1 to 3.1 times compared to the control, while with aeration it ranged from 1 to 6.3 times (Table 2). *Paenibacillus tundrae* IMV B-7915, *Pseudomonas yamanorum* IMV B-7916, *Paenarthrobacter* sp. 28-in-78, *Bacillus subtilis*, and *Streptomyces griseus* exhibited significant degradation activity on PHB films without aeration (Figure 3). Under additional aeration, the primary degraders of PHB were *Paenibacillus tundrae* IMV B-7915, *Flavobacterium* sp. 2B-in-99, *Bacillus mesentericus*, *Bacillus cereus*, and *Bacillus mycoides* (Figure 3, Table 2). Notably, aeration significantly enhanced the degradation of PHB biofilms by *Paenibacillus tundrae* IMV B-7915 and *Bacillus mycoides*, increasing the degradation by 4.9 and 6.3 times and the degradation rate by 27.1 and 34.6%, respectively, compared to control samples.

#### 3.1.2. Impact of the Cultivation of Microorganisms on PLA Degradation

PLA is a biodegradable plastic made from renewable resources like corn or sugarcane. It has several advantages, including being eco-friendly, compostable, and having a lower carbon footprint compared to traditional plastics. PLA is versatile and can be processed into different forms, such as films, fibers, and rigid structures. It is also biocompatible, making it safe for use in medical and food applications [60,61].

All the studied mold fungi degraded PLA films under static conditions (without aeration) (Figure 4). Compared to the control, the degradation levels ranged from 1.5 to 2 times, depending on the microorganism (Table 2). Additional aeration during mold cultivation did not significantly enhance the degradation rate. Only with the cultivation of *Penicillium chrysogenum* did the degradation of PLA films increase by 2.6 times under aerated conditions compared to control and was equal to 13%.

Among the bacteria tested, excellent results without aeration as well as with aeration showed *Bacillus subtilis* where the degradation of PLA films increased by 2.1 times at all studied conditions and was equal to 12% (Figure 4, Table 2). At this time the degree of degradation without bacteria was relatively high—approximately 6%—under both static and aerated conditions.

#### 3.1.3. Impact of the Cultivation of Microorganisms on the Degradation PHB/PLA Blend

Blending PHB, PLA, and PHA enhances mechanical and thermal properties, processability, and cost efficiency, creating versatile materials superior to pure polymers for diverse applications. Our work shows that polymer mixtures are degraded by microorganisms much worse when compared to ‘pure’ polymers (Figure 5, Table 2). Although, aerated samples degraded somewhat better than unaerated ones, of all the mold fungi tested, only in the case of *Penicillium chrysogenum* were there interesting results where the degradation of films increased by 1.4–1.6 times independent of conditions.

The studied bacterial species practically did not degrade PHB/PLA films, except for *Pseudoarthrobacter* sp. IMV B-7981 and *Flavobacterium* sp. 2B-in-99 for these the degradation under aeration was observed only. Compared to the control, the degradation was 1.7 times for *Pseudoarthrobacter* sp. IMV B-7981 and 1.5 times for *Flavobacterium* sp. 2B-in-99. The enhanced degradation of the PHB/PLA blend by *Pseudoarthrobacter* sp. IMV B-7981 and *Flavobacterium* sp. 2B-in-99 likely results from structural changes in the polymer matrix, synergistic enzymatic action at the polymer interface, and microbial metabolic adaptation to the mixed substrate. The presence of both polymers may create conditions that facilitate enzymatic hydrolysis, making degradation more efficient compared to pure PHB or PLA. In addition, the *Streptomyces griseus* showed a slightly higher ability to degrade PHB/PLA films than the control medium.

### 3.2. X-Ray Analysis of Biodegradation of Polymer: Impact of Weight Loss on Crystallinity

**Figure 6:**
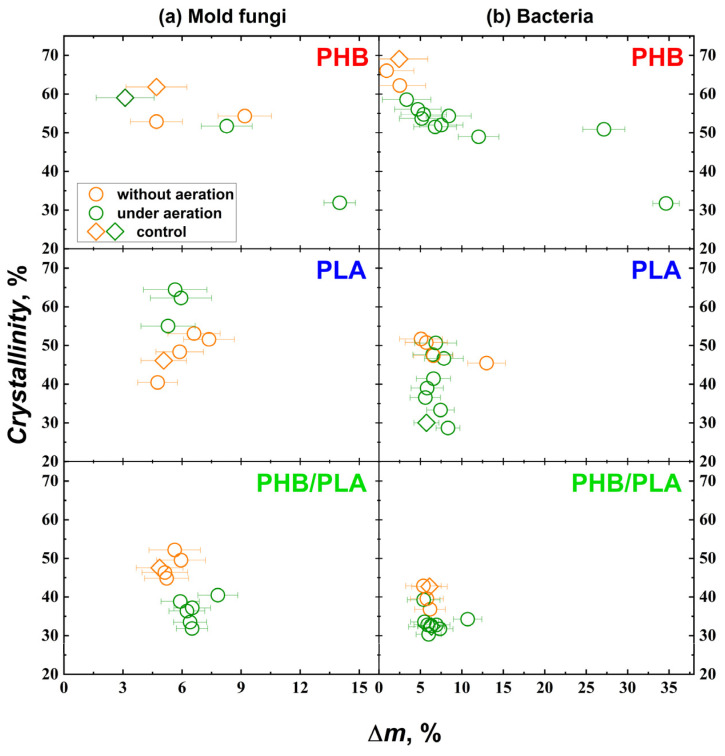
Correlation between crystallinity changes and polymer weight loss due to degradation by molds and bacteria for PHB, PLA, and PHB/PLA (62/38) blend.

XRD analysis provides valuable insights into polymer degradation by assessing changes in crystallinity. Unfortunately, it was not possible to analyze crystallinity changes in all samples. However, to explore the relationship between polymer film crystallinity and degradation rate (measured as polymer weight loss), we plotted the crystallinity values of all samples for which XRD spectra were obtained, distinguishing between fungal and bacterial degradation. Figure 6 illustrates the correlation between crystallinity changes and polymer weight loss resulting from degradation.

The crystallinity of PHB samples exposed to mold fungi ranged from 30% to 62%, with control samples incubated with or without aeration showing crystallinity values of nearly 60%. For PLA, the crystallinity ranged from 40% to 65%, with the control sample incubated without aeration exhibiting a value of 46%. For the PHB/PLA blend, the crystallinity ranged from 31% to 52%, with the control sample incubated without aeration at 47%. In samples degraded by bacteria, PHB crystallinity ranged from 31% to 66%, while the control sample incubated without aeration exhibited a crystallinity of approximately 68%. In our study, all investigated PHB samples exhibited a clear trend of decreasing crystallinity after incubation with microorganisms. This finding contrasts with previously published works, where a slight or pronounced increase in crystallinity was observed [62]. However, our results are in excellent agreement with those reported in [63], where a decrease in PHB crystallinity was noted after incubation under two different soil conditions.

For PLA, bacterial degradation resulted in crystallinity values between 36% and 51%, with the control sample incubated under aeration showing a crystallinity of 30%. For PHB/PLA blends, the crystallinity ranged from 31% to 42%, while the control sample incubated without aeration showed a value of about 32%. Notably, PHB crystallinity decreased in all samples compared to the control. For PHB, a clear trend of decreasing crystallinity with increasing weight loss was observed during both mold fungi and bacterial degradation. In contrast, PLA exhibited stable or increased crystallinity in samples degraded by molds and bacteria. These changes were independent of weight loss but were influenced by microbial species and incubation conditions. Several studies have shown that the crystalline regions of PLA are more resistant to degradation than the amorphous regions, and that the degradation rate decreases with increasing crystallinity [38,64,65]. Consequently, in most cases, PLA samples tend to show an increase in crystallinity during biodegradation. Notably, work [66] reported intriguing findings, demonstrating that crystallinity either increased or decreased within 14 days, depending on the type of enzyme used.

The reduction in crystallinity can be attributed to microbial species releasing plasticizing compounds that disrupt the organized structure of crystalline domains. Conversely, increased crystallinity is likely due to preferential degradation of the amorphous regions of the polymer. Alternatively, following the mechanism of stereoregular center enzymatic degradation, it is possible to consider that some microorganisms delivered such enzymes which are selectively able to split up biopolymer molecules only on the surfaces of crystalline entities.

The analysis of PHB/PLA blends appears to be less reliable because the peak intensity for PLA is significantly higher than for PHB. For PHB/PLA blends, samples degraded by mold fungi exhibited a notable reduction in crystallinity with minimal polymer mass loss. In contrast, bacterial degradation showed no consistent correlation between changes in crystallinity and polymer degradation. It is important to note that changes in the crystallinity of polymers can occur even when no weight loss is observed after cultivation. This suggests that structural transformations in the polymer are taking place, which will likely become evident over time.

### 3.3. Thermal Analysis of Biodegradation of Polymer

The thermal properties of the samples after degradation were studied using TGA, DTG and DTA analyses. Similar to the XRD analysis, only a limited portion of the samples was examined.

#### 3.3.1. Thermal Analysis of Biodegradation of PHB

Biodegradation of PHB samples was studied using control samples incubated in Sabouraud medium without microorganisms under aeration (degradation rate 4.1%) and PHB cultivated with *Trichoderma roseum* under aeration (degradation rate 17.5%). Similarly, samples incubated in TSB medium without microorganisms under aeration (degradation rate 5.5%) were compared to those cultivated with *Pseudoarthrobacter* sp. IMV B-7981 under aeration and *Streptomyces griseus* under aeration (degradation rates 5.2% and 5.4%, respectively).

**Figure 7:**
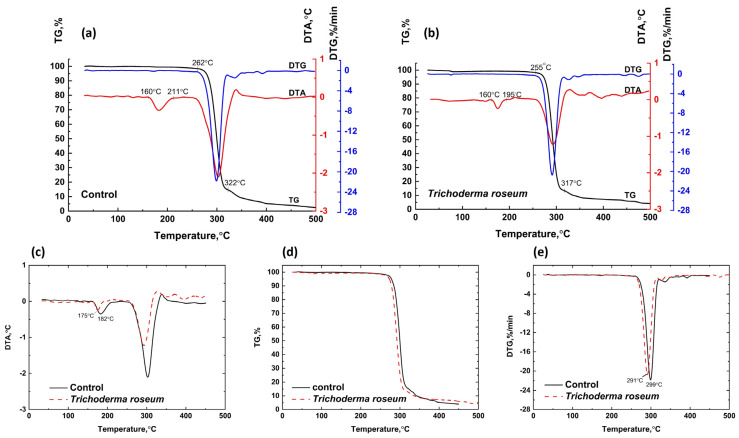
Combined TG, DTA, and DTG curves for PHB cultivated in Sabouraud medium under aeration (**a**) and PHB exposed to *Trichoderma roseum* under aeration (**b**), including DTA (**c**), TGA (**d**), and DTG (**e**) curves comparing the thermal characteristics of PHB in Sabouraud medium (black line) and PHB exposed to *Trichoderma roseum* (red line).

Figure 7 presents the TG, DTA, and DTG curves for PHB cultivated in Sabouraud medium and exposed to *Trichoderma roseum* under aeration. For the control PHB sample, an endothermic effect in the DTA curve at 160–211 °C corresponds to melting, without significant weight loss. The degraded sample shows a similar melting effect at 160–195 °C, but the thermal response is less pronounced, indicating lower heat resistance. The maximum melting temperature for the control sample is 182 °C, while it decreases to 175 °C for the degraded sample. A slight mass loss in the control sample (0.99%) within the temperature range of 211–262 °C and in the sample exposed to *Trichoderma roseum* (1.00%) within the range of 195–255 °C corresponds to the onset of initial degradation processes, accompanied by a deviation of the DTA curves into the endothermic region. In the temperature range of 262–322 °C, deep degradation occurs in the control PHB sample, marked by significant weight loss (83.75%), a sharp extremum on the DTG curve, and a pronounced endothermic effect on the DTA curve. Similar degradation processes are observed in the sample exposed to *Trichoderma roseum* within the range of 255–317 °C, accompanied by an intensive weight loss (84.96%).

The PHB sample degraded by mold fungi shows lower thermal stability than the control sample, as indicated by the earlier onset of degradation and the extremum shift on the DTG curve to lower temperatures. The extremum of the control sample is at 299 °C, while the extremum for the sample exposed to *Trichoderma roseum* is at 291 °C. The combustion of residual degradation products occurs in the temperature ranges of 322–500 °C and 317 °C, respectively, with both samples showing gradual weight loss and the appearance of exothermic effects on the DTA curves.

**Figure 8:**
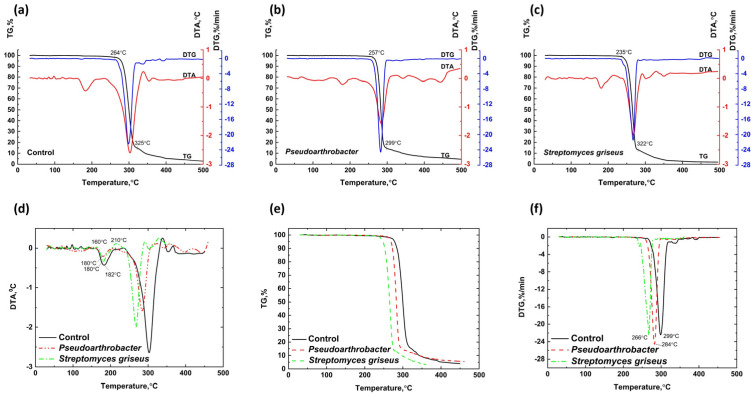
Combined TG, DTA, and DTG curves for PHB cultivated in TSB medium under aeration (**a**) and PHB exposed to *Pseudoarthrobacter* sp. IMV B-7981 (**b**) and *Streptomyces griseus* (**c**) under aeration, including DTA (**d**), TGA (**e**), and DTG (**f**) curves comparing the thermal characteristics of PHB in a TSB medium (black line) and a PHB sample exposed to *Pseudoarthrobacter* sp. IMV B-7981 (red line) and *Streptomyces griseus* (green line).

Figure 8 presents the TG, DTA, and DTG curves for PHB cultivated in TSB medium without aeration and exposed to *Pseudoarthrobacter* sp. IMV B-7981 under aeration and *Streptomyces griseus* without aeration. On the DTA curves within the temperature range of 160–210 °C, an endothermic effect is observed, corresponding to the melting of the samples. Notably, the thermal effects of the samples exposed to *Pseudoarthrobacter* sp. IMV B-7981 and *Streptomyces griseus* are less pronounced than those of the control sample, and their peaks are shifted to lower temperatures, indicating reduced heat resistance in the degraded samples. The peak of the endothermic melting effect for the control sample is observed at 182 °C, while for the samples exposed to *Pseudoarthrobacter* sp. IMV B-7981 and *Streptomyces griseus*, this peak occurs at 180 °C.

In the temperature range of 210–264 °C, initial degradation processes begin in the control PHB sample developed in the bacterial environment, characterized by slight weight loss and a deviation of the DTA curve toward endothermic effects. Similar processes are observed in samples exposed to bacteria, occurring within the temperature ranges of 210–257 °C and 210–235 °C, respectively. Above 270 °C, intensive degradation processes start in the control PHB sample. Comparable degradation begins at 257 °C for the sample exposed to *Pseudoarthrobacter* sp. IMV B-7981 and at 235 °C for the sample exposed to *Streptomyces griseus*. These degradation processes are accompanied by pronounced endothermic effects on the DTA curves and peaks at lower temperatures on the DTG curves.

PHB samples degraded by bacterial influence exhibit lower thermal stability than the control PHB sample, as evidenced by shifts in the onset temperatures for degradation and the DTG curve peaks to lower temperatures. Specifically, the degradation peak for the control sample occurs at 299 °C, while the peaks for samples exposed to *Pseudoarthrobacter* sp. IMV B-7981 and *Streptomyces griseus* are observed at 284 °C and 266 °C, respectively. This reduced thermal stability in the degraded samples is likely due to the presence of degradation products. At temperatures exceeding 325 °C for the control sample, 299 °C for the sample exposed to *Pseudoarthrobacter* sp. IMV B-7981, and 280 °C for the sample exposed to *Streptomyces griseus*, combustion of the residual decomposition products occurs in both the control and degraded PHB samples, accompanied by exothermic effects on the DTA curves.

In all cases where microorganisms act on PHB, a slight decrease in polymer crystallinity is observed, influencing its thermal stability and melting point. A similar finding was reported in [63], where PHB samples degraded under two different soil conditions. Interestingly, even in cases where the degradation rate under the influence of microorganisms was comparable to or slightly lower than that of the control sample (e.g., the effects of *Pseudoarthrobacter* sp. IMV B-7981 and *Streptomyces griseus* on PHB under aeration), a marked decrease in thermal stability and melting point was detected. This suggests significant structural degradation of the polymer macrochains, although the integrity of the polymer sample and its overall mass remain largely unaffected at this stage.

#### 3.3.2. Thermal Analysis of Biodegradation of PLA

Biodegradation for PLA samples was studied using a control sample after incubation in the Sabouraud medium without microorganisms and under aeration (degradation rate nearly 5.1%) as well as in the Sabouraud medium at cultivation with *Trichoderma roseum* under aeration where the degradation rate was equal to 6.6%. In turn, biodegradation for PLA samples was studied using a control sample after incubation in the TSB medium without microorganisms and under aeration (degradation rate nearly 5.8%) as well as in TSB medium at cultivation with *Pseudoarthrobacter* sp. IMV B-7981 and *Streptomyces griseus* under aeration where degradation rate was equal to 7.4% and 5.7%, respectively.

**Figure 9:**
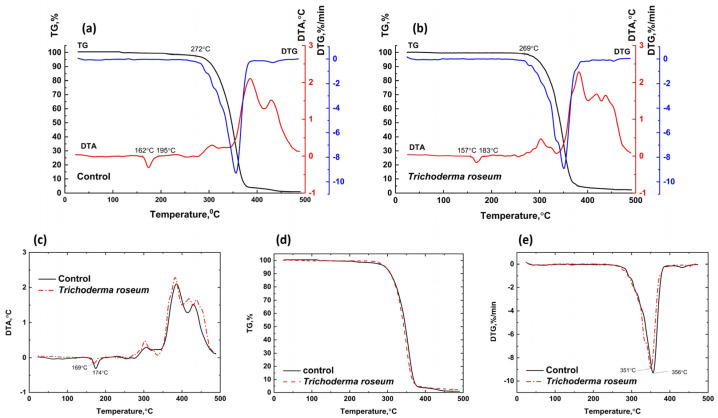
Combined TG, DTA, and DTG curves for PLA cultivated in Sabouraud medium with aeration (**a**) and PLA exposed to *Trichoderma roseum* under aeration (**b**), including DTA (**c**), TGA (**d**), and DTG (**e**) curves comparing the thermal characteristics of PLA in Sabouraud medium (black line) and PLA exposed to *Trichoderma roseum* (red line).

The results of the TG, DTA, and DTG analysis for PLA incubated in a Sabouraud medium with aeration and a PLA sample exposed to *Trichoderma roseum* under aeration are presented in Figure 9.

A slight weight loss (0.89%) observed in the temperature range of 20–162 °C for the control PLA sample incubated in Sabouraud medium under aeration corresponds to the release of volatile components. Similarly, for the PLA sample exposed to *Trichoderma roseum*, a release of volatile compounds (0.55%) occurs within the range of 20–157 °C. This behavior is associated with deviations in the DTA curves of the PLA samples toward endothermic effects.

In the control PLA sample, an endothermic effect on the DTA curve between 162 and 195 °C corresponds to the melting of PLA incubated in Sabouraud medium, without any associated weight loss. By contrast, the melting of the sample exposed to *Trichoderma roseum* appears as an endothermic effect on the DTA curve within the range of 157–183 °C. Compared to the control, this effect is less pronounced, with its peak occurring at 169 °C shifted to a lower temperature range than the peak in the control sample, which appears at 174 °C. This suggests a slightly reduced crystalline phase content in the sample exposed to *Trichoderma roseum*.

For the control sample, a gradual weight loss (1.80%) observed between 195 and 272 °C, along with a DTA curve deviation toward exothermic effects, reflects the onset of initial destructive and thermo-oxidative processes in PLA. In contrast, these processes for the sample treated with *Trichoderma roseum* occur over the range of 183–269 °C, corresponding to a smaller weight loss of 0.64%.

At temperatures above 272 °C, active destructive and thermo-oxidative processes begin in the control PLA sample, culminating in the combustion of residual decomposition products, as evidenced by exothermic effects on the DTA curves. In the sample exposed to *Trichoderma roseum*, these processes initiate slightly earlier, at 269 °C, and show a more intense weight loss (Figure 9c) compared to the control, indicating marginally reduced thermal stability after mold fungal exposure.

Further evidence of this reduced thermal stability is the shift of the DTG curve extremum to a lower temperature (351 °C) in the degraded sample compared to the control sample (356 °C). Finally, the combustion of the residual products in the sample exposed to *Trichoderma roseum* generates a more pronounced exothermic effect on the DTA curves relative to the control sample (Figure 9e).

**Figure 10:**
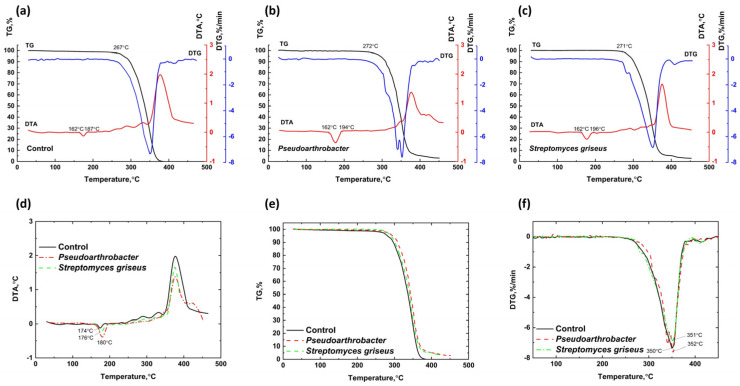
Combined TG, DTA, and DTG curves for PLA cultivated in TSB medium without aeration (**a**) and PLA exposed to *Pseudoarthrobacter* sp. IMV B-7981 (**b**) and *Streptomyces griseus* (**c**) under aeration, including DTA (**d**), TGA (**e**), and DTG (**f**) curves comparing the thermal characteristics of PLA in a TSB medium (black line) and a PHB sample exposed to *Pseudoarthrobacter* sp. IMV B-7981 (red line) and *Streptomyces griseus* (green line).

The results of the TG, DTA, and DTG analysis for PLA incubated in a TSB medium without aeration and PLA samples exposed to *Pseudoarthrobacter* sp. IMV B-7981 under aeration and *Streptomyces griseus* without aeration are presented in Figure 10. A minor weight loss of the PLA samples in the 20–162 °C temperature range indicates the release of volatile components. This loss is associated with a deviation of the DTA curve into the endothermic region. In the control PLA sample, an endothermic effect observed in the 162–187 °C range corresponds to the melting of PLA cultivated in a bacterial medium, with no significant weight loss. For the samples exposed to *Pseudoarthrobacter* sp. IMV B-7981 and *Streptomyces griseus*, endothermic melting effects appear on the DTA curves within the 162–194 °C and 162–196 °C ranges, respectively. Compared to the control sample, these endothermic effects are more pronounced, with peaks shifted to higher temperatures, indicating a higher crystalline phase content in these samples. Specifically, the endothermic peak for the PLA sample exposed to *Pseudoarthrobacter* sp. IMV B-7981 occurs at 174 °C, while for the sample exposed to *Streptomyces griseus*, the peak appears at 180 °C. A gradual weight loss (1.26%) observed in the control sample over the 187–267 °C range, along with a shift in the DTA curve to the exothermic region, signifies the onset of initial destructive and thermooxidative processes in PLA. For the samples exposed to *Pseudoarthrobacter* sp. IMV B-7981 and *Streptomyces griseus*, these processes occur within 194–272 °C and 196–271 °C, respectively, with reduced weight losses of 0.98% and 1.06%, suggesting a higher thermally stable component in the degraded samples. Above 267 °C, active destructive and thermooxidative processes commence in the control PLA sample, culminating in the combustion of decomposition residues, accompanied by exothermic effects on the DTA curves. In the samples exposed to *Pseudoarthrobacter* sp. IMV B-7981 and *Streptomyces griseus*, these destructive processes begin slightly later, at 272 °C and 271 °C, respectively, indicating enhanced thermal stability compared to the control. This is further supported by the shift of the onset and DTG curve extremum temperatures to higher ranges. The DTG extremum for the control PLA sample occurs at 359 °C, while for samples exposed to *Pseudoarthrobacter* sp. IMV B-7981 and *Streptomyces griseus*, the extrema are at 351 °C and 352 °C, respectively. The combustion of decomposition residues in PLA samples exposed to bacteria resulted in less intense exothermic effects on the DTA curves compared to the PLA control sample.

PLA samples exposed to bacteria (*Pseudoarthrobacter* sp. IMV B-7981 and *Streptomyces griseus*) exhibited slightly increased thermal stability and melting points compared to the control sample, indicating lightly enhanced crystallinity. In contrast, PLA samples exposed to *Trichoderma roseum* showed a slight decrease in thermal stability and melting point. Thermal analysis of PLA biodegradation during composting, as presented in [67], indicated a decrease in the mobile amorphous phase throughout the process. Unlike PHB samples, where even the absence of wight loss after exposure to microorganisms demonstrated a distinct pattern of property changes, the changes in PLA were less pronounced. This suggests a slower degradation process for PLA under microbial influence compared to PHB.

## 4. Conclusions

The search for microorganisms capable of efficiently degrading plastics is critical due to increasing environmental pollution. This study examined the biodegradation of PHB, PLA, and their composite films by 11 bacterial and 6 mold fungal species, including Antarctic strains.

All polymer samples showed some degradation in a nutrient medium, even without microorganisms. Aeration significantly enhanced PHB degradation, particularly by mold fungi and certain bacteria. *Aspergillus oryzae* exhibited the highest PHB degradation efficiency under aeration, while *Aspergillus awamori* was most effective in non-aerated conditions. Bacterial degradation of PHB increased twofold under aeration, with *Paenibacillus tundrae* IMV B-7915 and *Bacillus mycoides* showing the highest activity.

PLA degradation by mold fungi was generally low, except for samples cultivated with *Penicillium chrysogenum* under aeration, where degradation was significantly higher. Among bacteria, *Bacillus subtilis* showed the highest PLA degradation under both aerated and non-aerated conditions. PHB/PLA composite films degraded more slowly than pure PHB or PLA but showed the highest degradation with *Penicillium chrysogenum* (both aerated and non-aerated) and with *Pseudoarthrobacter* sp. IMV B-7981 and *Flavobacterium* sp. 2B-in-99 under aerated conditions.

Structural and thermal analyses confirmed these findings. XRD revealed a decrease in PHB crystallinity corresponding to weight loss, while PLA exhibited stable or increased crystallinity, depending on microbial species and conditions. Thermal analysis showed a slight reduction in PHB thermal stability after exposure to *Trichoderma roseum*, *Pseudoarthrobacter* sp. IMV B-7981, and *Streptomyces griseus*, consistent with XRD data. Conversely, PLA samples exposed to *Pseudoarthrobacter* sp. IMV B-7981 and *Streptomyces griseus* exhibited increased thermal stability, while *Trichoderma roseum* slightly reduced PLA stability.

These findings highlight the potential of specific bacterial and fungal species, particularly from extreme environments, in promoting effective polymer degradation. Further studies on enzymatic mechanisms and environmental applications of these strains could advance sustainable plastic waste management strategies.

## Figures and Tables

**Table 1:** Culture of the microorganisms and condition of cultivation used in the study.

№	Microorganism	Conditions for Cultivating		Remarks
		Without Aeration (wa)	Aeration (a)	
Mold species (Sabouraud medium)				
Control		+	+	
1m.	*Aspergillus oryzae*	+	+	
2m.	*Penicillium chrysogenum*	+	+	
3m.	*Trichoderma lignorum*	+	+	
4m.	*Aspergillus niger*	+	+	
5m	*Aspergillus awamori*	+	+	
6m.	*Trichothecium roseum*	+	+	
Bacterial species (Trypticase Soy Broth)				
Control		+	+	
1b.	*Paenibacillus tundrae* IMV B-7915	+	+	from Antarctic sourses
2b.	*Pseudomonas yamanorum* IMV B-7916	+	+	from Antarctic sourses
3b.	*Paenarthrobacter* sp. 28-in-78	+	+	from Antarctic sourses
4b.	*Pseudoarthrobacter* sp. IMV B-7981	+	+	from Antarctic sourses
5b.	*Flavobacterium* sp. 2B-in-99	+	+	from Antarctic sourses
6b.	*Bacillus mesentericus*	+	+	
7b.	*Bacillus megaterium*	+	+	
8b.	*Bacillus cereus*	+	+	
9b.	*Bacillus mycoides*	+	+	
10b.	*Bacillus subtilis*	+	+	
11b.	*Streptomyces griseus*	+	+	

**Table 2:** Degradation (times) of polymer films by microorganisms compared to control obtained in same conditions.

The Type of Mold Fungi	PHB		PLA		PHB/PLA	
	Without Aeration	Aeration	Without Aeration	Aeration	Without Aeration	Under Aeration
**Mold Fungi Species**						
*Aspergillus oryzae*	≈1	6.6	1.6	1.2	1.1	1.3
*Penicillium chrysogenum*	1.1	3.8	1.5	2.6	1.4	1.6
*Trichoderma lignorum*	2.2	3.4	2.0	1.5	1.2	1.4
*Aspergillus niger*	1.4	2.0	1.7	≈1	1.1	1.2
*Aspergillus awamori*	4.0	2.3	2.0	1.2	1.1	1.4
*Trichothecium roseum*	2.1	4.3	1.6	1.3	1.2	1.3
**Bacteria species**						
*Paenibacillus tundrae* IMV B-7915	2.0	4.9	1.1	1.1	1.0	≈1
*Pseudomonas yamanorum* IMV B-7916	2.5	1.4	1.1	1.1	1.0	≈1
*Paenarthrobacter* sp. 28-in-78	2.5	1.2	1.0	1.3	≈1	≈1
*Pseudoarthrobacter* sp. IMV B-7981	≈1	≈1	1.1	1.3	≈1	1.7
*Flavobacterium* sp. 2B-in-99	≈1	2.2	≈1	1.4	≈1	1.5
*Bacillus mesentericus*	1.1	1.5	1.0	1.0	1.0	≈1
*Bacillus megaterium*	1.4	≈1	1.1	1.0	≈1	1.1
*Bacillus cereus*	1.2	1.8	1.0	0.9	≈1	1.0
*Bacillus mycoides*	1.0	6.3	1.0	1.2	≈1	≈1
*Bacillus subtilis*	1.7	0.9	2.1	2.1	≈1	≈1
*Streptomyces griseus*	3.1	1.0	0.9	1.0	1.4	1.2

Note: In some case, as a result of errors in the studies, calculated values were weakly less 1 and this values were written as ≈1.

## Data Availability

The original contributions presented in this study are included in the article. Further inquiries can be directed to the corresponding author.
